# 

**Published:** 1881-01

**Authors:** 


Vol. I. JANUARY, 1881. No. I.

THE

### HOMŒOPATHIC PHYSICIAN:

A MONTHLY JOURNAL OF MEDICAL SCIENCE.

*"Magna est veritas et praevalebit."*

E. J. LEE, M. D.

EDITOR.

